# Aqueous Humor Liquid Biopsy Identifies Murine Double Minute 4 Segmental Gain in Retinoblastoma: Implications for Chemotherapy Response and Precision Oncology

**DOI:** 10.1200/PO-25-00250

**Published:** 2025-08-20

**Authors:** Elaine Huang, Shreya Sirivolu, Venkata Yellapantula, Prachi Kale, Peter Kuhn, James Hicks, Brianne Brown, Heidy Paniagua, Drishti Pandya, Rachana Shah, Jesse L. Berry, Liya Xu

**Affiliations:** ^1^ The Vision Center at Children's Hospital Los Angeles, Los Angeles, CA; ^2^ USC Roski Eye Institute, Keck School of Medicine, University of Southern California, Los Angeles, CA; ^3^ Center for Personalized Medicine, Children's Hospital Los Angeles, Los Angeles, CA; ^4^ The Saban Research Institute, Children's Hospital Los Angeles, Los Angeles, CA; ^5^ Norris Comprehensive Cancer Center, Keck School of Medicine, University of Southern California, Los Angeles, CA; ^6^ USC Michelson Center for Convergent Biosciences and Department of Biological Sciences, Los Angeles, CA; ^7^ Division of Hematology-Oncology, Department of Pediatrics, Cancer and Blood Disease Institute, Children's Hospital Los Angeles, University of Southern California, Keck School of Medicine, Los Angeles, CA

## Introduction

Retinoblastoma (RB) is a pediatric intraocular malignancy arising from cone precursor cells in the retina because of biallelic inactivation of the *RB1* tumor suppressor gene.^1^ Historically, in vivo molecular information was not available for RB tumors because of contraindication to direct tumor biopsy.^2^ To address this, we developed aqueous humor cell–free DNA (cfDNA) liquid biopsy, enabling real-time tumor-derived cfDNA analysis at diagnosis and throughout therapy.^3,4^ This approach allows for routine identification of genomic alterations for in vivo RB tumors, including rare RB subtypes with uncommon genetic features.

Aqueous humor cfDNA (AH-cfDNA) liquid biopsy enables identification of molecular markers such as the p53 regulator murine double minute 4 (*MDM4*), known to be amplified in multiple cancers and associated with apoptosis regulation and cancer progression. While chromosome 6p gain and focal *MYCN* amplification have been associated with high-risk disease,^5,6^ focal *MDM4* alterations on 1q are not routinely described for RB at diagnosis. We highlight *MDM4* among the coamplified genes at chromosome 1q32.1 because of its recurrent amplification in RB and biologically validated role in promoting disease progression and potential treatment resistance in studies on RB tumor tissue from enucleated eyes.^1,7,8^ Other coamplified genes in this region, while of biological interest, lack functional or clinical evidence supporting a role in RB pathogenesis or therapeutic response. Our analysis thus highlights *MDM4* as a more robust and substantiated candidate for risk stratification using AH-cfDNA.

*MDM4*, like its homolog *MDM2*, inhibits *TP53*-mediated responses to DNA damage, a key pathway for chemotherapy-induced cell death. Although *TP53* mutations are rare in RB, overexpression of *MDM4* may suppress p53 function, potentially contributing to treatment resistance. This raises the possibility that *MDM4* amplification may define a biologically and therapeutically distinct RB subset.

Here, we present the first known case of an RB tumor with a segmental copy number gain peaking at *MDM4*, detected in AH at diagnosis and monitored using the AH longitudinally for therapeutic response. This case links segmental *MDM4* gain to poor response to melphalan but sensitivity to topotecan, suggesting possible distinct therapeutic vulnerabilities in *MDM4*-associated RB. These findings highlight the impact of the AH-cfDNA liquid biopsy as a powerful tool for precision oncology and real-time treatment response monitoring in RB, with potential for genomic-driven treatment selection.

## Case Report

This research was conducted under the Institutional Review Board approval at Children's Hospital Los Angeles (CHLA). Written informed consent, including consent for publication and use of images, was obtained from the participant's parents before inclusion in the study. Treatment decisions were blinded from AH genomic test results; AH data were kept separately from clinical data until the final retrospective analysis.

**FIG 1.**
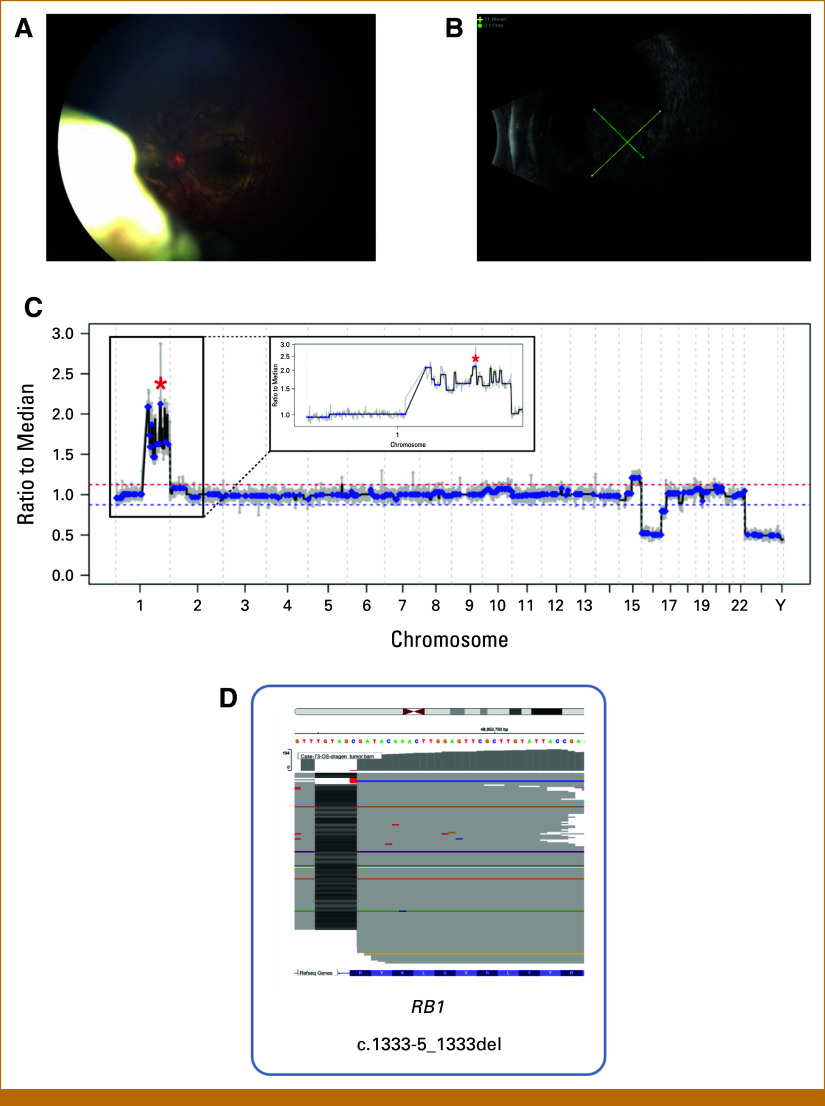
(A) Diagnostic fundus photograph of the left eye demonstrated a largely predominantly endophytic tumor forming in the anterior nasal retina. (B) B-scan ultrasonography of the left eye showed an elevated retinal-based dome-shaped intraocular lesion with scattered diffuse intralesional calcium with measurements of 11.86 × 8.67 mm. (C) SCNA profile reflected a focal amplification of *MDM4* on 1q32 with more than four copies, with no evidence of 6p gain and *MYCN* amplification. (D) *RB1* mutational analysis of AH ctDNA from the left eye identified c.1333-5_1333del mutation, with a VAF of 97.72%. Created using BioRender.com. Huang, E. (2025) BioRender.com. MDM4, murine double minute 4; SCNA, Somatic Copy Number Alteration; VAF, variant allele frequency.

A 35-month-old male presented with a several-week history of leukocoria and was diagnosed with unilateral RB at CHLA. At diagnosis, the intraocular pressure measured 18 mmHg in the right eye (OD) and 15 mmHg in the left eye (OS). Fundoscopic examination of OS revealed a predominantly endophytic tumor in the anterior nasal retina (Fig 1A). The macula and optic nerve were unaffected, and the retina remained attached. Vitreous seeding was observed in two quadrants, consisting of both spherical and cloud-like seeding. The eye was classified as International Intraocular RB Classification (IIRC) Group D/American Joint Committee on Cancer Stage cT2b, a category that typically accounts for more than 50% of RB eyes at diagnosis.

B-scan ultrasonography of OS identified a dome-shaped intraocular lesion with scattered intralesional calcium, measuring 11.86 × 8.67 mm (Fig 1B). No tumor was detected in OD. Germline testing did not reveal *RB1* germline or pathogenic mutations in any other genes, and there was no family history of RB.

AH was collected by clear corneal paracentesis and analyzed for copy number alterations and somatic variants using shallow whole-genome sequencing and targeted sequencing as previously described.^9^ AH was taken at diagnosis and throughout treatment; analysis of diagnostic AH demonstrated a segmental copy number gain at *MDM4* on 1q32 (Fig 1C), with no evidence of chromosome 6p gain or *MYCN* amplification. Tumor fraction (TFx), the proportion of sampled cfDNA that is tumor-derived, was assessed from each AH sampling^10^; the TFx at diagnosis was 98.33%. Targeted sequencing of AH detected a somatic *RB1* c.1333-5_1333del mutation as the pathogenic variant with loss of heterozygosity (LOH; Data Supplement, Fig S1). A single *TP53* variant, c.215C>G (p.P72R), was also identified; this common polymorphism is not considered pathogenic.

**FIG 2.**
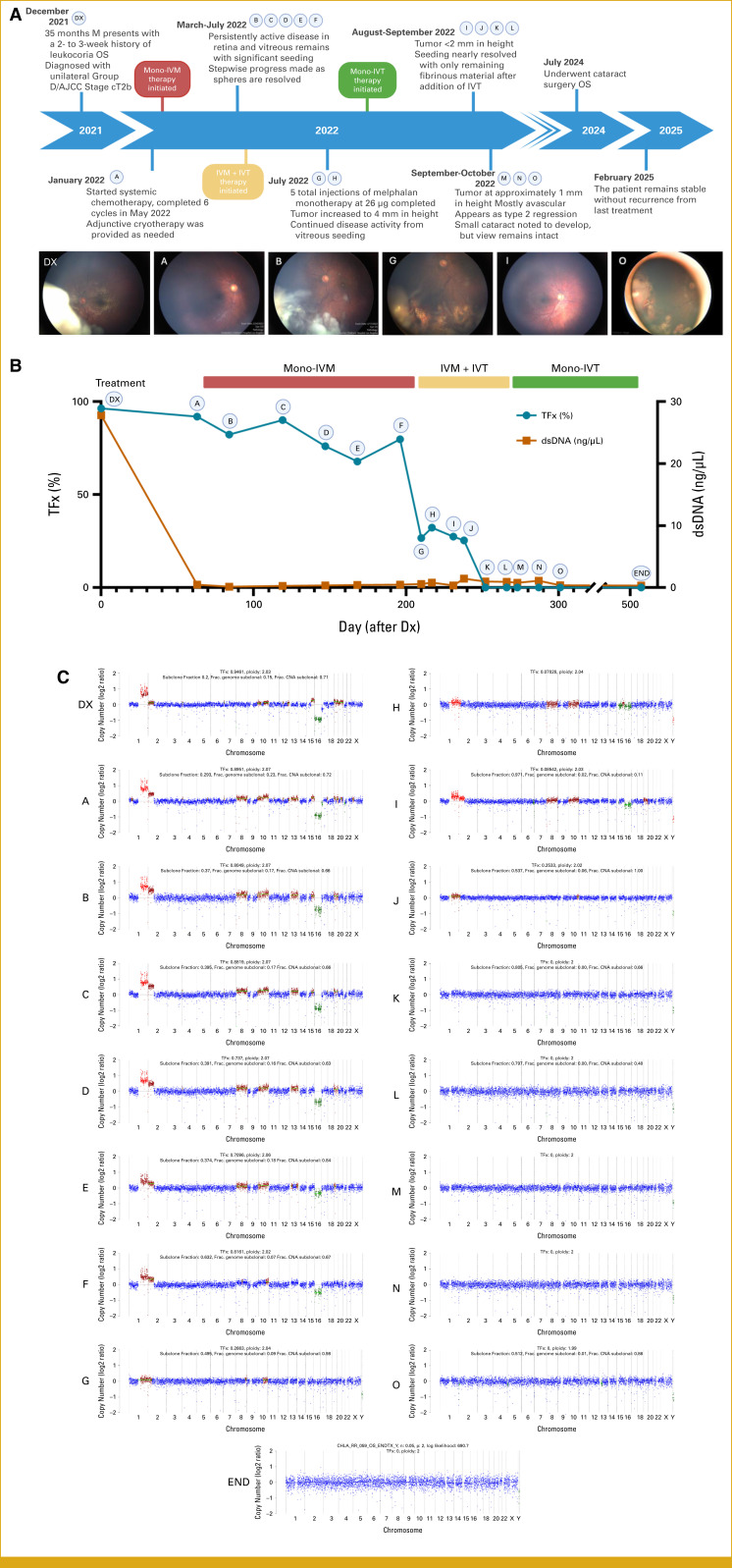
(A) Treatment timeline and corresponding fundus images at critical time points; (B) Molecular profile of TFx and dsDNA concentration throughout the treatment regimen; (C) Corresponding ichorCNA profiles. A full set of all fundus images is presented in the Data Supplement (Fig S2). Created using BioRender.com. Huang E (2025). IVM, intravitreal melphalan; IVT, intravitreal topotecan; TFx, tumor fraction.

The patient received systemic intravenous chemotherapy with carboplatin, etoposide, and vincristine (CEV) at a hospital closer to their home, with monthly examinations under anesthesia at CHLA. Despite retinal tumor regression after two cycles of CEV, vitreous seeding persisted. Three months postdiagnosis, laser consolidation and intravitreal melphalan (mono-IVM) at 26 mcg for each cycle were added before cycle 3. Spherical seeds regressed after the first mono-IVM injection (Fig 2A, Image B), but a retinal nodule persisted despite six total CEV cycles including local consolidation with cryotherapy, laser, and five mono-IVM. Most residual tumor volume consisted of diffuse vitreous seeding in two quadrants, with an inferior cloud of seeding.

Because of persistent vitreous seeding after five mono-IVM injections, dual intravitreal therapy with melphalan (26 mcg/cycle) and topotecan (IVT, 30 mcg/cycle) was initiated. After the first IVM + IVT injection, dust seeds persisted, but spheres and the cloud resolved. The TFx decreased notably from 79.74% to 26.63% (Fig 2B) with no observed toxicity. By the fourth injection, seeding had nearly resolved, leaving only fibrinous material. TFx, which remained elevated during mono-IVM, significantly decreased after six dual injections. Transitioning to topotecan monotherapy, the patient received four additional IVT doses. After 15 total injections, seeding regressed, with calcified fibrinous material and a smaller avascular nodule (0.7 mm) resembling type 2 scarring. There was no further clinically significant tumor-derived cfDNA detected in the final AH (0%), indicating complete response. The patient developed a cataract requiring posterior chamber intraocular lens implantation but remained stable without recurrence at a 27-month follow-up.

## Discussion

Melphalan and topotecan are the most commonly used intravitreal chemotherapeutic agents for RB. Melphalan was the first described^11^ and is often considered standard first-line treatment for RB vitreous seeding, whereas intravitreal topotecan is typically reserved for refractory cases, eyes with melphalan toxicity or monocular cases given the lower risk of toxicity.^12^ Requiring 15 cycles of intravitreal chemotherapy, incorporating both drugs, as in this case with persistent seeding, is unusual. The median number of intravitreal injections for vitreous seeding in RB rarely exceeds five.^13^ In addition, the decision to start or stop therapy is often based solely on clinical examination. Here, molecular evaluation of TFx corroborated persistent tumor activity seen clinically.

This case highlights a rare and aggressive RB subtype with *MDM4* segmental amplification, identified for the first time by AH-cfDNA liquid biopsy at diagnosis. While *MDM4* amplification has been reported in RB tumor tissue^1,5,6,14-16^ (Table 1), its clinical relevance in treatment response remains unclear. A retrospective review of previous work^15^ identified two additional cases with *MDM4* gains (Data Supplement, Fig S3): one that failed IVM therapy and required secondary enucleation and the other that underwent primary enucleation. These cases suggest a potential, though not yet definitive, association between *MDM4* gain and differential treatment response.

**TABLE 1.** Literature Review of *RB* and Focal *MDM4* Gene Amplification

Author	Year	Cancer Type	Sample Type	Genomic Technology	Information Summary
Fenghua Yu et al^6^	2019	RB	Primary RB tumor tissue	SNP genotyping by rtPCR	MDM4 *rs11801299* and *rs1380576* polymorphisms associated with increased risk of developing RB, increased tumor invasion, and poor pathologic differentiation
Armin R. Afshar et al^5^	2020	RB	Primary RB tumor tissue, peripheral blood	NexGen sequencing	Focal amplification of MDM4 in RB associated with higher histologic grade and anaplasia
Keita Togashi et al^17^	2020	RB	RB cell culture	Immunoblot analysis	CEP1347 reduces expression of MDM4, therefore activating the P53 pathway and having an antiproliferative effect on RB cells
Jessica Le Gall et al^18^	2021	RB	Peripheral blood, primary enucleated RB tumor tissue, aqueous humor	NexGen sequencing	MDM4 SNPs *rs11801299* and *rs1380576* susceptibility factors for RB and SNP *rs11801299* demonstrated as biomarkers of tumor aggressiveness. CEP 1347 and nutlin-3a activate P53 in RB cells by reducing MDM2/MDM4 overexpression
Helen R. Davies et al^14^	2021	RB	Primary RB enucleated tumor tissue, peripheral blood	Whole-genome sequencing	Focal amplification of MDM4 identified in one RB tumor sample of 21

Abbreviations: MDM4, murine double minute 4; RB, retinoblastoma.

Melphalan remains highly effective in most RB cases as it induces DNA cross-links that accumulate beyond a tolerable threshold, leading to tumor cell death. However, this case demonstrates that not all RB tumors respond to melphalan. The presence of *MDM4* amplification and breakage-fusion-bridge (BFB)–driven chromosomal instability may confer resistance to melphalan.^16,19^ Instead, this tumor responded much better to topotecan, a topoisomerase I inhibitor that induces replication stress—a mechanism potentially more effective in genomically unstable tumors.^12^

*MDM4*, a negative regulator of *TP53*, can impair p53-mediated apoptosis, undermining the cytotoxic efficacy of DNA-damaging agents like melphalan. Functional suppression of *TP53* may thus contribute to drug resistance, even in the absence of pathogenic *TP53* mutations. In this case, the only TP53 variant identified was c.215C>G (p.P72R), supporting the interpretation that *TP53* was functionally intact but suppressed. Topotecan, by inducing p53-independent replication stress, may circumvent this suppression, explaining the tumor's selective sensitivity.

While based on a small number of cases, these findings are biologically plausible and clinically meaningful. Further studies should examine whether *MDM4* amplification correlates with p53 pathway impairment and whether restoring p53 function—through *MDM2/MDM4* inhibition—can resensitize tumors to melphalan. Integrating molecular profiling into RB management could help guide therapy, particularly in high-risk or refractory cases.

Emerging clinical trials targeting the *MDM2/MDM4*–p53 axis underscore the therapeutic relevance of this approach. Agents such as Nutlin-3a and ALRN-6924,^20^ designed to restore p53 activity by inhibiting *MDM4* and *MDM2* interactions, have shown promise in preclinical RB models. Such strategies may offer targeted benefit in *MDM4*-amplified tumors with suppressed but genetically intact *TP53*. Stratifying tumors by *MDM4* status could help identify candidates for these therapies and inform the development of personalized treatment regimens.

This case represents the first clinical observation linking segmental 1q gain at *MDM4* to differential chemotherapy response in RB. It supports a shift from phenotype-driven to genotype-guided therapy, using real-time genomic surveillance through AH-cfDNA liquid biopsy. Early identification of molecular markers like *MDM4* may enable precision treatment, reduce ineffective interventions, and improve outcomes for aggressive RB subtypes.

## Data Availability

A data sharing statement provided by the authors is available with this article at DOI https://doi.org/10.1200/PO-25-00250. The data presented in this study will be available on request from the corresponding author. Due to NIH funding, the data will be available to other researchers via NIH GDS/dbGAP controlled databases and will also be available to the public upon request from the corresponding author.
